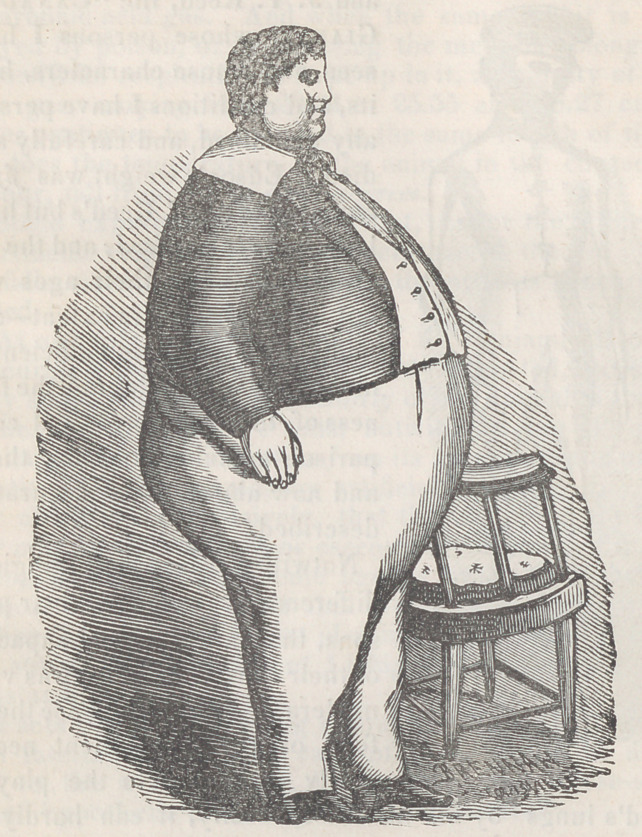# On Vital Organic Chemistry

**Published:** 1852-08

**Authors:** C. Caldwell

**Affiliations:** Late Professor of the Institutes of Medicine in the University of Louisville


Art. IV.—*On Vital Organic Chemistry.* By C. Caldwell, M. D., Late Professor of the Institutes of Medicine in the University of Louisville.

[This article was intended as a Report to the American Medical Association, but its venerable author was unable to attend the meeting of the Association, and his views are submitted to the profession through the pages of this *Journal.*]—*Editors Western Journal of Medicine & Surgery.*

At a meeting of the American Medical Association, at

Cincinnati, May, 1850, the following resolution was adopted:

"*Resolved*, That Dr. Caldwell be requested to take into consideration the subject of *Vital Organic Chemistry*, and report to the next meeting, whether, in his judgment, it can be justly called a *branch of science*, and, if so, how far a knowledge of it can be rendered available to the welfare of man."

Were it not for one obstacle, the preparation of this Report would be a humble and comparatively an easy task. But that hindrance renders it complex and difficult.

The obstacle alluded to is the difficulty of ascertaining a correct and satisfactory knowledge of the subject of the report, apprehending and definitely comprehending the precise import of the phrase "*Vital Organic Chemistry*."

This is not less than the twentieth—perhaps than the fiftieth time I have written, lectured, debated, or in some way exercised my mind in the consideration of the same subject. And on each repetition of the exercise, I have endeavored in vain to acquire a knowledge of the interpretation applied by the members of the chemico-physiological sect of the medical profession, to the expression "vital organic chemistry." For it is to that sect alone that the expression belongs. By the vital sect it is disowned as *an error*, or a *nullity*. To explain myself more fully on the subject.

In the year 1844, when engaged in a controversial correspondence on this topic, I proposed to my antagonist for solution, the following plain and pointed interrogatories:

"What is the genuine meaning of the phrases "*Animal Chemistry*," "*Vegetable Chemistry*" or "*Vital Organic Chemistry*?" For I presume the import of the three forms of expression is the same. The attribute of *vitality* must be involved in each of them. For vegetables and animals are equally *vital*.

"Do the advocates of the doctrines of animal, vegetable, and vital organic chemistry contend, that, in the *formation*

of living animal and vegetable tissues and secretions, the forces called *chemical affinities* or *attractions* and *repulsions* take part, as positive and efficient agents, in like manner as they do, in the modification of various sorts of dead matter? In the formation, for example, of a leaf, a blossom, or any sort of fruit; and in the formation of any animal fluid, tissue, or organ, of the blood, the bile, the urine, a muscle, the eye, the kidney, the liver, the brain, or any other kind of organized and living animal or vegetable matter—is it contended, that, in the formation of these products, the chemical forces act as they do in the formation of granite, marble, siliceous crystal, sulphuric acid, sulphate of iron, or any other sort of dead matter belonging exclusively to the mineral kingdom?"

Such are the interrogatories, which, to ascertain, if practicable, the meaning attached to the phrase "vital organic chemistry," by the chemico-physiological sect in medicine, I proposed through the press, eight years ago; and though the production containing them has been read by multitudes, they remain unanswered. Nor do I think it probable that they will ever be reduced to a different condition, by any of the members of the chemico-physiological sect. Nor are those gentlemen without a reason I suspect for remaining silent. An answer, whatever might be its spirit or matter, might be shown to be prejudicial to either their hypothesis, their own judgment or consistency, or to the three combined.

But an explanation, real or reputed, of the phrase in question, I must receive from some source, else it will be impracticable for me to proceed with my report; because I cannot rationally report or comment in any way on that to which I am a stranger. I shall therefore endeavor to derive some portion at least of the intelligence I need from the writings of Professor Liebig, who bears to the chemico-physiological sect the high and authoritative relation of Grand Master. His *dictum* on the subject, I shall on that

2

account regard as authentic. From his celebrated work entitled "Animal Chemistry," or "Organic Chemistry," I extract the following passages, and could extract dozens more tending to precisely the same purport.

"In the animal body we recognize as the *ultimate cause of all force*, only *one cause*, the CHEMICAL ACTION, which the elements of the food, and the oxygen of the air exercise on each other. The only known ultimate cause of *vital force*, either in animals or in plants, is a CHEMICAL PROCESS. IF THIS BE PREVENTED, THE PHENOMENA OF LIFE DO NOT MANIFEST THEMSLLVES. * * * * All vital activity arises from the mutual action of the oxygen of the atmosphere and the elements of the food."

"Physiology has sufficiently decisive grounds for the opinion, that every motion, every manifestation of force, is the RESULT OF A TRANSFORMATION *of the structure or of the substance of the animal body.*"

[But that motion or transformation, I reply, is effected, not by a chemical force; but by a superior force, which *controls* that of chemistry, and commands, modifies, and adapts to its own nature the transformation and its effects.—*Au.*]

"In the processes of nutrition and reproduction, we perceive the passage of matter from a state of motion to that of rest, (static equilibrium.) Under the influence of the nervous system, this matter enters again into a state of motion. The *ultimate* causes of these different conditions of the *vital force* are *chemical forces.*

"The power to effect transformations does not belong to the *vital principle.* Each transformation is owing to a disturbance in the attraction of the elements of a compound, and is consequently a *purely chemical process.*

"The combinations of the chemist relate to the change of matter, *forward and backward to the conversion of food into the various tissues and secretions, and to the irmetamorphosis in lifeless compounds.*"

[The only common sense construction of this passage is, that the same forces or powers of action, which at one time produce a *living*, at another produce a *dead* form of matter. In other words, that there is no difference between a *living* and a *dead result*, and therefore, that *difference* and *identity*, *opposition* and *concurrence* are the same.—*Au.*]

"How beautifully and admirably simple, with the aid of these" (*chemical*) "discoveries appears the process of *nutrition* in animals, the formation of their organs, &c.

"The self-regulating steam-engines furnish no unapt image of what occurs in the animal body.

"The animal body, in regard to the production of *heat* and *force*, acts like one of these engines.

"The carbon of the blood, which is converted into carbonic acid within the body, must give out exactly as much heat as if it had been burned *in the air*, or in oxygen gas.

"If chemical action be excluded, as a condition of *nervous agency*, it means nothing else than to derive the presence of *motion, the manifestation of force, from nothing.*

"The most decisive experiments of physiologists have shown, that the process of *chymification is independent of the vital force*; that it takes place in virtue of a purely chemical action, exactly similar to those processes of *decomposition and transformation*, which are known as PUTREFACTION, FERMENTATION, OR DECAY."

[Had I time to comment on this passage, I would undertake to establish decisively three points, both interesting and important:

*First.* That chymification is not a *chemical*, but a *vital* process.

*Second.* That no vital function or form of action bears the slightest resemblance to putrefaction or decay. And

*Third.* That, instead of benefiting genuine physiology by his experiments on St. Martin, Dr. Beaumont has seriously injured it.—*Au.*]

"The power of elements to unite together, and to form peculiar compounds, which are generated in animals and vegetables, is CHEMICAL AFFINITY.

"We should not permit ourselves to be withheld, by the idea of a VITAL PRINCIPLE, from considering, in a CHEMICAL POINT OF VIEW, the process of TRANSFORMATION of the food, and its ASSIMILATION by the various organs.

"We know that an organized body cannot *generate* substances, but only change the mode of their combinations, and that its SUSTENANCE and REPRODUCTION depend upon the CHEMICAL transformation of the matters which are employed in its nutriment, and which contain its own constituent elements. Whatever we regard as the causes of these transformations, the act of transformation is a PURELY CHEMICAL PROCESS."

Such are the extracts I have derived from Professor Liebig's Animal Chemistry. And, as heretofore mentioned, I could derive from it, I know not how many more of *the same import.* And its author is of course, as already stated, the oracle of his sect, whose responses and dictates few of his followers venture to contest.

Now, although these extracts do not of themselves amount to a real definition or even interpretation of the expression, Vital Organic Chemistry; they include the elements, out of which one may be constructed. They show conclusively, that Professor Liebig, however he may occasionally express himself, on the subject of a vital principle, considers and virtually declares every process, whether general or special, that is performed in the living system of man and other animals, and in vegetables also, to be performed exclusively by *chemical agency.* They make it clearly appear, that he regards both the *composition and decomposition*, the *building up* and *pulling down* of living organic matter, as a *purely chemical process.*

By chemistry alone, according to his teaching, is the food of man and other animals converted into chyme, the chyme into chyle, the chyle into blood, and the blood into nerve muscle, bone, and every other tissue, as well as into bile, saliva, urine, cutaneous perspiration, and every other secretion and excretion of the system. In truth, from the showing and acknowledgement of the Professor himself, not only, according to his creed of human construction, is man originally formed by chemical agency; he is subsequently, in his whole economy, as exclusively governed and kept in play by it, as is a "*self-regulating steam engine.*"

Nor, under the light of a rigid and correct analysis of the subject, is this representation less applicable to his *mental* than to his *corporeal functions.* Chemistry is virtually made to give birth, modification, and sustenance to the whole of them.

For if there be in existence a more unscrupulous system of materialism than that contained in Liebig's Animal Chemistry, I know not where it is to be found.

While one sacred writer declares man to be, in his compound capacity, "fearfully and wonderfully made;" and another that he is 'made but a little lower than the angels,' the Professor of Giessen, in his account of him, makes him appear but little higher in his nature and being, than a German stove, or a Belgian beer-barrel! Had not experienee and observation therefore abundantly taught me, that true christianity and religious partyism of a ferocious cast are necessarily in a state of belligerency toward each other, it would surprise me to find in the train of his followers and defenders scores of sulphur-lipped fanatics and bigots.

Nor is Liebig the only celebrated chemico-physiologist that represents man as a chemical product. Muller, Roget, Prichard, Carpenter and others of the same school, have maintained the same notion.

Muller, in a special manner, asserts that the seminal or generative substances which the male and the female contribute toward the immediate production of the embryo

(the *semen masculinum* to be planted and the *feminine ovariaas the soil* to receive and nourish it) to be "*produced from the proximate compounds of the blood, by a chemical process.*"

On this ground the embryotic ingredients contributed, compounded, and constructed, by the generative process, are also of necessity chemical. And so must be the issue they jointly engender. Nor, in compliance with an irreversible law of nature, can the *chemical embryo*, thus begotten and sustained, fail to grow up into *a chemical man.* So utterly ludicrous is the web of false doctrine, in which every thorough-going chemico-physiologist necessarily entangles himself! And this is as applicable to Roget, Carpenter, and Prichard, as it is to Muller.

I am now prepared to enter on the business of my report; because I now know respecting the subject of it all that I am at present destined to know.

From the contents of the writings of Liebig, Muller, and the other chemico-physiologists referred to, concurring with sentiments I have myself long entertained, and often expressed, I shall define Vital Organic Chemistry to be the groundless notion that—

*Genuine chemistry can and does, without the cooperation and aid of any other force or principle of action, generate and sustain living organic matter, in the forms and capacities of vegetables and animals.*

True, when the advocates of this notion speak of it in general and theoretic terms, they connect with it a superior controlling and plastic force, which they denominate *vital.* But when they describe specially and practically any function of living organized beings, they refer its production to a force *purely chemical.* And, singular as it may appear, it is notwithstanding true, that several, if not the whole of them, when pressed on the subject, pronounce the superior and plastic additional force to be the immediate influence of the WILL OF THE CREATOR---an act or rather an artifice amounting in them to an abandonment of reason and science, and the adoption, as a substitute, of an expedient of rank superstition and fanaticism. It is virtually proclaiming every function of animals and vegetables to be something *above the operation of natural causes,* and *therefore miraculous*—all which shall presently more fully appear. Meantime, from the considerations on the subject hitherto adduced, I am inclined to report, that,

*In my judgment, Vital organic chemistry cannot be justly called a branch of science. It is a* MEANINGLESS NOTION.

And this report I shall endeavor further to substantiate by conclusive arguments. To execute this measure the more effectually, my first act shall be, to attempt the refutation of the most distinguished chemico-physiologists, who, in their writings on the subject under consideration, have offered sentiments contradictory of mine. And the most effectual mode of overthrowing an opponent being to set him irreconcilably at war with himself; my design is to refute my antagonists, by arguments derived from their own productions on the topic I am considering. In other more express and forcible terms, I shall, by making it clearly and conclusively appear, that their own arguments (if such they may be called) are self-destructive, I shall affix on them the felony of *literary suicide.* And, as the most prominent and anthoritative of the corps, I shall begin with Professor Liebig. In his work on Animal Chemistry, I find the following passages, which will be perceived to be directly opposed in sentiment to those which have been just quoted from the same volume.

"A rational physiology cannot be founded on mere reaction; and the living body cannot be viewed as a *chemical laboratory.*

"With all its discoveries, modern chemistry has performed but slendor services to physiology and pathology.

"In the animal ovum, as well as in the seed of a plant, we recognize a certain force, the source of growth, or increase in the mass, and of reproduction or of supply of matter consumed. * * * This force is called the ***vital force***, the *vis vitæ*, or *vitality*.

"He is not the true chemist, who endeavors to apply to the animal organism his notions derived from purely chemical *processes*. He has not the remotest intention of undertaking the explanation of any really ***vital phenomenon*** on *chemical principles*.

"In what form, or in what manner the vital force produces mechanical effects in the animal body, is altogether unknown, and is as little to be ascertained by experiment as the connexion of chemical action with the phenomena of motion.

"So it is with vital force, and with the phenomena exhibited by living bodies. The *cause* of these phenomena is not *chemical force*; it is not electricity or magnetism. It is a *peculiar force*, because it exhibits manifestations which are formed by *no other known force*.

"The vital force appears as a moving force or cause of motion, when it *overcomes* the chemical forces, cohesion, and affinity, which act between the constituents of food * * * * The vital force is manifested as a cause of motion in *overcoming* the chemical attraction of the constituents of food, and is also the cause which ***compels them to combine in a new arrangement***."

Though I could adduce, from Animal Chemistry, scores of other passages to the same purport with the foregoing, the labor would be superfluous. Those already quoted more than neutralize—they utterly *demolish* the work, as a chemico-physiological production. To prove that a publication is *self-inconsistent, is to destroy its reputation and influence*—the penalty thus irrevocably inflicted on Professor Liebig's book.

I shall now expose in the same way, to the same extent, and on the same subject, the errors of Dr. Roget and Dr. Carpenter.

"However," says the former of these distinguished writers, the laws which regulate vital phenomena may appear, on a superficial view, to differ from those by which the physical changes taking place in inorganic matter are governed, still there is really no essential difference between them." * * "It may, in like manner, be contended, that the affinities which hold together the elements of living bodies, and which govern the elaboration of organic products, *are the same* with those which preside over inorganic products."

So much for that gentleman's views, as a chemico-physiologist, in relation to the production and maintenance of living organic matter. Let us now contemplate his views on the same subject, as a vitalist, compare them with those just stated, and judge of the contrast.

"The foundations," he says, "of the edifice" (of the chick in the egg) "are laid in the homogeneous jelly, *by the efforts of the vital powers.*" "At first," he continues, "all the energies of *vitality* are directed to the raising of the fabric, and to the extension of those organs, which are of greatest immediate utility; but still having a prospective view to farther and more important ends," and so on to the close of the process, the whole work of developing, fashioning, and locating the fœtal organs being assigned exclusively, to the "*efforts of the vital powers,*" and to the "*energies of vitality;*" *chemistry having, from beginniug to end, no agency in the work.* In another part of the same publication, Dr. Roget correctly asserts, that the products of the *vital powers*, in their *formative processes,* are uniformly *organs, designed and fitted for the performance of given purposes;* while the products of chemical forces are uniformly both *organless* and *designless.*

And, for a further distinction equally essential, between vital and chemical products, he might have correctly added, that the former are uniformly bounded by curve or waving lines; and the latter by straight lines and angles.

Dr. Carpenter, whose ability and celebrity as a writer, neither need commendation from the pen of a friend, nor dread censure from that of a foe; when under the influence of his chemico-physiological mood, writes as follows:

"Reason has been already given for the belief, that the affinities which hold together the elementary particles of organized structures are not different from those concerned in the inorganic world; and it has been shown that the tendency to *decomposition*" *(putrefaction)* "after death, bears a very close relation with the activity of the *changes that take place in the part during life*."

But, when in the mood of a vitalist, which occasionally visits him, the same gentleman writes to the following purport:

"Organization, and vital properties are simultaneously communicated to the germ by the structures of its parents. Those vital properties confer upon it the means of itself assimilating, and thereby organizing and endowing with vitality, the materials supplied by the inorganic world."

And in the same spirit he further says:

"The agency of vitality does not change the properties of the elements" (of the food and the blood) "but simply combines them *in the modes which we cannot imitate*."

The late Dr. Prichard, one of the most keen, close, and elaborate thinkers and writers on matters of medicine that England has produced, was several years ago a bold and thorough-going chemico-vitalist. But a further examination of the subject now under our consideration, induced him so far to alter and modify his views, that, in one of his later publications, he thus expressed himself:

"We may, if we choose to do so, term the cause which governs the organization and vital existence a *plastic prin-*

*ciple;* but it is a principle *endowed with intelligence* and *design.* It is in fact nothing more than the ENERGIES OF THE DEITY. The development of generie, specific, and individual forms, as well in the vegetable, as the animal kingdom, can be accounted for only, by ascribing it to the universal energy and wisdom of the Creator."

Yet was the Doctor, I say, at one time, a firm believer in the doctrine (or rather *notion*) of *chemical vitality.* But having, from his own superior penetration and sagacity, become thoroughly convinced of the fallacy and preposterousness of that hypothesis, and perceiving the absolute necessity that a plastic power and not a mere power of *chemical affinity* must be employed in the construction of living organized matter—under these impressions, actuated by some unaccountable crotchet, which, at times, takes possession of the most distinguished minds, instead of looking for the requisite plasticity in a *secondary* cause; he referred it at once to the GREAT CAUSE of causes; thus representing the development of even a blade of grass or a leaf of mucor as a *miraculous event!*

Having thus shown that the leading chemico-physiological writers referred to have, by self-contradiction, refuted themselves, in relation to the subject under consideration, I shall endeavor to strengthen that refutation, by a few arguments drawn from my own resources. And the matter of those resources will be derived from the conditions and manifestations of the human body in a living state.

But prevented, by a want of time, from including in my discussion of the subject, all the functions of the system of man, and making it appear that a belief in the existence of vital organic chemistry, as a branch of science, is forbidden by the nature and economy of the whole of them; I shall confine my remarks principally to one of the most prominent and important of them, and the attribution of whose *origin* and *continuance* to the influence of chemical forces involves less absurdity than a similar notion respecting the attributes of any other sort of vital action.

There are especially two of the functions belonging to the system of man and other warm blooded animals, which constitute at present the chief topics of contention between the vital and the chemico-physiological sects of the medical profession. They are *calorification* and *chymification.* And I am in my own unqualified opinion, prepared to show, beyond denial or doubt, that neither of them is explicable on chemical principles. But as I cannot, in my consideration of the question before me, embrace them both, I shall select the former. And Liebig being, as already stated, the leader of the sect opposed to me, I shall confine my remarks principally to his dogmas on the function to be considered. Should I satisfactorily establish the insufficiency of them, my work will be done. He being vanquished, his followers may be expected to surrender at discretion.

The fundamental assumption of the Professor is, that the heat of the human body and of the bodies of other similarly organized animals, like the heat of a German stove, is the result of combustion. And in this he is correct; except that, in the former, carbon and hydrogen are both burned; but in the latter carbon perhaps alone. Another of his dogmas is, that the same amount of carbon, whether burned *in* the human body, or *out* of it, always evolves the same amount of caloric, and produces of course the same degree of heat. But in this opinion I shall endeavor to show hereafter, that he is incorrect. I shall unquestionably show, that, if his opinion be not, in all respects, essentially unfounded, it certainly involves at least one very striking phenomenon utterly inconsistent with the principles he advocates.

In his fundamental propositions and his comments on them toward the establishment of his doctrine of animal heat, he has laid down one in particular, and contended for its validity, which I must specially notice; because it is, in my judgment, as groundless as it is important—being, in both respects, of a high order.

That their bodies may retain the requisite temperature, it is necessary for the inhabitants of the frigid zones, especially during the winter season, to generate a much greater amount of heat, than it is for those of the temperate and the torrid zones. To enable them to do this, Liebig contends, that in the former region, persons possessing chests and lungs of the same capacity, inhale, in a given time (because the atmosphere around them is colder) a much greater quantity of oxygen, than do those of the latter region, the atmosphere around them being much warmer.

This hypothesis, though plausible, and very extensively, if not universally believed, I pronounce utterly erroneous. And my reasons for thus characterizing it, are so clear and substantial, that I deem it impossible for any one of even common ability of mind to fail, on due consideration of them, to acknowledge their perspicuity as well as their truth. In testimony of this I shall briefly detail them.

In each climate, torrid, temperate, and polar (and this Professor Liebig himself admits) the same quantity of air makes its way into the lungs, in the same space of time. And he farther correctly admits, that by the time the air has reached the interior of the lungs, its temperature is the same (99 or 100° of Fahrenheit) whatever may be the temperature of the surrounding atmosphere from which it is derived. And I myself, (on the ground of experiment) positively add, that however cold may be the atmosphere from which it is extracted, the air inspired has attained the acme of its temperature (99° or 100°) even before it has passed the *rima glottidis.*

No matter then from what atmosphere it is drawn (polar, temperate, or tropical) if the air inspired (being the same in volume) possesses, when it enters the bronchiæ and their air-cells, the same temperature, it contains also the same amount of oxygen ; which again can unite with and burn up only the same quantity of carbon and hydrogen. Nor are these all the identities that characterize it which the occasion urges me to notice.

With entire truth and confidence may I add, that (in direct contradiction and overthrow of the assertion of Liebig) whatever may be the temperature of the incumbent atmosphere, the air drawn from it, by inspiration, possesses when in the lungs the same degree of humidity.

This assertiou I make to encounter and defeat the design and aim of the allegation by Liebig, that, in the warm atmosphere of the tropics, moisture occupies and fills up the place of oxygen in the cold and dry atmosphere of the polar region.

Admit the truth of this position as respects the air *without* and *around* the persons of the inhabitants, it is not true of the air in their lungs. As already stated, the air in the bronchiæ and air-cells is always of the same temperature, whether the weather be warm or cold ; and the secretion from the internal surface of the lungs maintains it in the same degree of humidity, whether the weather be moist or dry.

Did the Professor of Giessen moreover understand meteorology as well as he ought to understand it, before undertaking to excogitate a new doctrine in it, he would not need to be told that the driest condition the atmosphere ever attains exists when the harmattan wind blows. And that prevails in its high degree only in the torrid zone, and chiefly, if not solely during the hottest season of the year.

It is not true therefore, that the tropical atmosphere contains less oxygen than the polar, because it superabounds in humidity. It contains less *when* and *because* it is more expanded by its more elevated temperature. For the amount of oxygen in a cubic foot, yard, or other measure of the atmosphere is always and necessarily (other things being alike) in precise accordance with such expansion. And in the lungs of a living and healthy human being unadulterated atmospherical air is uniformly in the same condition.

The truth of this position, if it be held in doubt, I freely stake on the following experiment.

Let the ablest analytical chemist of the day, (Professor Liebig himself,) during ordinary weather, take from each of two bodies of the atmosphere, one of them at the temperature of zero, and the other at that of 80° of Fahrenheit, a cubit foot of air. Let him heat these two extracted portions to the temperature of 100°. From each of them again let him take an equal quantity by weight or measurement, and accurately analyze it, and he will find in each the same amount of oxygen, and of water in the form of vapor. For, come from what climates they may, equal quantities of atmospheric air, of the same temperature with each other, possess equal quantities of oxygen, as well as of nitrogen and carbonic acid gas, and also of humidity under similar circumstances—of humidity I mean in the air-cells of the lungs.

In asserting therefore that individuals possessed of chests and lungs of the same size, and breathing an equal number of times in a minute, inspire, in a given space more, oxygen in cold climates than in warm—in Archangel than in Calcutta,—Professor Liebig has committed a mistake, which he ought publicly to renounce, and no longer mislead his credulous followers. Yet does the *vital* process of respiration *(because it is vital)* evolve, from an equal quantity of calorific materials, a greater amount of caloric in the winter atmosphere of Labrador, than it does in the winter atmosphere of Panama; or in that of any part of tropical Africa. And this I say it does under the controling influence of a principle or force of a higher order and character (I might say of a higher order of *being*) which enables it to produce the effect—that effect being indispensable to the economy of living matter. Nor is this the only error, respecting animal heat, of a nature somewhat similar, into which that Professor has fallen.

Because infants possess lungs more voluminous, in proportion to the size of their systems, than adults do, the Professor has asserted that they also evolve by respiration (in the same proportion) more caloric, and therefore possess a higher temperature.

Let the validity of this notion be tested by the following experiment, and I venture to pledge my experience and observation that it will be found wanting.

Expose to a cold and humid atmosphere, under a covering equally light and scanty, an infant and an adult alike healthy and robust for their age, (each remaining equally quiet) and mark the issue. Before the adult will be, in any perceptible degree affected, the temperature and pulse of the infant will begin to decline. On this point unerring instinct correctly informs the inferior animals. Hence the attention and care with which the mother protects her young from the cold. And for want of attention similarly affectionate and wise, thousands of infants suffer and perish. This clearly shows that the quantity of heat evolved is not, in all cases, proportionate to the qnantity of oxygen employed in respiration. And to me the following facts appear to testify to the same purport.

The chest and lungs of some adults are much more capacious, in proportion to the size of their whole systems, than those of others; and they inspire as fully and frequently. They inhale, of course, in a given time, a greater amount of oxygen; and neither their perspiration nor any other discharge by them is more copious. Yet is their temperature no higher.

On the ground of his own experiments, we are assured by Treviranus, a writer of reputation and credibility, that, in an atmosphere at the temperature of 80½° Fahrenheit, the honey bee exhales three times as much carbonic acid gas, as it does in an atmosphere at the temperature of 50½°. Yet, may we confide in the doctrine of Professor Liebig, and other chemists, the insect inspires, in the latter case, the greatest amount of oxygen; and we do not know, nor have we any reason to believe, that it consumes, in its aliment, less carbon. Nor when the atmosphere is at the temperature of 50½° is the honey bee at all impaired, or in any way wanting in the functions of vitality.

Muller informs us, on the authority of experiments he has performed or witnessed, that some cold-blooded animals inhale *three times* as much oxygen as is requisite to form the amount of carbonic acid gas which they discharge. Will Professor Liebig, or any of his followers inform us, how the superabundant quantity is disposed of by those animals?

Muller again informs us that man exhales a greater amount of carbonic acid gas when the barometer is *low* than he does when it is *high*. Yet does the air contain, in a given volume, a larger portion of oxygen in the latter case, than it does in the *former*, and of each of its other ingredients also a higher ratio.

The human female expires a larger quantity of carbonic acid gas when in a state of pregnancy, than when in an unimpregnated one. Yet does she inhale less oxygen, especially during the latter stages of her pregnancy, on account of the diminished expansion of the lungs, owing to the augmented pressure on them by the enlargement of the uterus.

These facts are derived from the economy of animated nature, in a healthy condition. Let us attend to the phenomena exhibited by it, in the same respect, in a state of disease.

The temperature of a patient, in an advanced stage of typhus fever, when he is greatly debilitated, is often as high as 105° or 106° of Fahrenheit, and, at times higher. Yet is his respiration exceedingly restricted on account of debility. He inhales, in a given time, less oxygen by a third or fourth part, than he does in health ; and from its emaciated condition, his body is equally deprived of carbon.

In cases of asthma and hydrothorax the same is true. Respiration is preternaturally confined, and the temperature at times, though not perhaps always, preternaturally high.

The following passages, on the same subject, are extracts from my "Physiology Vindicated ;" a work published in the year 1843 ; and no refutation or exposure of their errors, possessing the least merit of any description that has ever been attempted.

"But it is more especially by the phenomena of phthisis pulmonalis that the hypothesis of our author" (that now under consideration) "is irretrievably overthrown. When sufferers under that complaint are able to breathe with but a small portion of a *single lung*, when they have not in their entire systems an ounce of fat to furnish carbon, and but comparatively little water to furnish hydrogen ; and when the amount of their food and drink (especially the former) is exceedingly small, and none of it oily—under this destitution of all materials essential to combustion—as well of oxygen to consume as of "fuel" to be consumed —they manifest, for weeks and even months, a high degree of febrile temperature, amounting to 105° or 106° of Fahrenheit—while it should, according to the hypothesis we are examining, be far below the human temperature of health. Were there no other argument but this, to urge against our author's notion, that the vital temperature is the result of *common combustion*, it would alone completely demolish it."

Scores of cases of phthisis pulmonalis, of this description, have fallen under my own observation. In corroboration of their truth, the following very extraordinary case of the same complaint (not seen however by myself) is referred to, in a pamphlet, which I published in the year 1844. I here extract a brief account of it, from that production.

The case is recorded on the authority of Graves and Stokes of Dublin, and appears, in the 5th volume of the Dublin Hospital Reports. It is also referred to, in the American Journal of Medicine, vol. 8, year 1831, p. 218, and is as follows:

"Each lobe of the lungs was extensively tuberculated; the pulse beat 126 times in a minute; and the temperature was *highly febrile*. Yet did the patient respire but *fourteen times* in a minute!"

To contend that the temperature of this patient, under this case of disease, was produced by the *common combustion* of carbon and hydrogen, I pronounce preposterous! As well may a disputant exercise his sophistry, in contending for an effect without a cause, or for the existence of two adjacent hills without a hollow!

I well know that, by some of my opponents, it is contended that, in these cases of inordinately *augmented* febrile heat and *diminished* respiration, the excess of temperature is attributable to the *want* of perspiration. But I as well know that the *cause alleged* is far too *limited and feeble* to account for the magnitude of the effect produced. And a fact strongly analogous to this I shall demonstrate by *count and admeasurement*, which, when accurately executed, never deceive.

Nor is the following representation less irreconcilable with the dogma of Professor Liebig. I quote again from a publication of my own, which has also lain uncontradicted, since the year 1844.

"Sir Benjamin Brodie ascertained by experiments, whose accuracy is not doubted, that a rabbit full grown and healthy, expires every half hour, 28 22 cubic inches of carbonic acid gas. And when the same rabbit is destroyed by poison, or by bisecting the medulla oblongata, and artificial respiration is kept up in it, a quantity of the same gas, varying from 20.24 to 25.55 and 28.27 cubic inches continues to be exhaled in the same length of time. Yet does the temperature of the animal in the course of an hour, fall from *six to eight degrees.*

"Some experimenters assert that, under the influence of artificial respiration, the temperature of the dead animal falls more rapidly, than when the process is not employed.

"At any rate, the experiments of Sir Benjamin Brodie, if accurately performed, and correctly reported, satisfactorily show, that when the *vitality* of an animal is extinguished, its temperature falls, notwithstanding the production *in* and the elimination *from* its system, of the usual quantity of carbonic acid, by artificial respiration. And they also show conclusively, that the vital forces, whatever may be their nature or essence, are capable of maintaining animal heat by some agency other than that of *chemical combustion.* And that therefore the economy of a living human body is not identical with that of a German stove, the authority of Liebig to the contrary notwithstanding.

"Facts like the foregoing I could continue to detail almost interminably. And each of them would be alike fatal to the wild, dashing, ill-concerted and worse-supported hypothesis of Liebig and his followers."

But I shall content myself for the present with a statement of the following experiments and the observations accompanying them, which are exclusively my own. As far as my information and belief on the subject extend, the first conception and performance of them were mine. Should their disclosure and record therefore eventuate in usefulness and merit, the issue will pass to my credit. And in case the reverse be their lot, I shall without a murmur submit to the blame.

I here exhibit to the reader the figures of two extraordinary men; Calvin Edson, the "WALKING SKELETON," and J. T. Reed, the "CANADIAN GIANT," whose persons I have seen, and whose characters, habits, and conditions I have personally examined, and carefully studied. Edson's weight was *fifty-seven pounds*, and Reed's but little less than *six hundred;* and the difference between their ages was but inconsiderable in extent--certainly far from being sufficient to mar in any way or degree, the fairness of the experiment and comparison made when I saw them, and now about to be accurately described.

Notwithstanding the prodigious difference in the size of their persons, that in the natural capacity of their chests and lungs was very moderate. In consequence therefore, of the impediment necessarily presented to the play of Reed's lungs by his immense obesity, it can hardly be doubted that his respiration was inferior in compass to the respiration of Edson. Even a presumption that it *might* have been *superior* is *inadmissible.* Let it be conceded however, that the quantities of oxygen they inhaled, in a given time, were equal—as they inspired with about equal frequency.

In neither the kind nor amount of their diet and drink was there any material difference. And of their secretions and excretions the same is true. Singular and perhaps incredible as, to some persons, the fact may appear, there was no authentic evidence, that Edson discharged from his system, through any of its outlets, a greater portion of the inherent ingredients of it, than Reed did from the same outlets of his. In a special manner, there was no evidence that he discharged more by *perspiration.*

It may be said therefore, without any material departure from fact, that the two individuals, of whom I am speaking, conveyed into their systems, through the trachea and the œsophagus, the same kind and quantity of matter, solid, fluid, and aeriform. To imitate the language of Liebig—the same amount of *fuel* to be consumed, and the same amount of oxygen to consume it, for the purpose of heating their "*body stoves*," was regularly appropriated to that end. Nor was there perceptibly a greater escape of their product from one of them than from the other.

But the difference in the amount of matter, of which those stoves consisted was immense—and fatty or oily matter deposited in the apparatus of Reed excepted, the kinds were the same. Nor *did I then, nor do I now* venture even to conjecture, from what source such a huge mass of oleagenous matter was derived. But *there* it was; and *there* was also the skin and bone fabric of Edson extracted from a similar repository.

It was necessary for Edson to raise his stove, which weighed but little more than fifty pounds to the temperature of 100° Fahrenheit; and he did so; and there the process was checked. And it was equally necessary for Reed to heat his stove, which weighed near six hundred pounds, to the same temperature, else ruin must ensue. And he performed the deed by the same extent of combustion, as his means of action. But, to drop metaphor, and resume directness of speech. With a given amount of oxygen, carbon, and hydrogen Reed raised to 100° of Fahrenheit, the temperature of near *six hundred* pounds of *living human substance;* while, with the same amount, Edson raised to the same height, *but fifty-seven pounds.* Working therefore with the same means, Reed surpassed Edson, in operation and effect, *more than tenfold.*

Such I say was the case, while the bodies of the two individuals were living. But suppose them to be lifeless and cold. In that condition, diffuse through the carcass of Reed the quantity of caloric generated by Edson while living, and through the carcass of Edson the quantity evolved during life, by the system of Reed. What will be the effect? The answer is equally plain and easy. By the operation, the dead body of Reed will not be raised in temperature *ten degrees;* while the chief portion of that of Edson will be *reduced to cinders*—all in it that is exhalable being turned into gas. Suppose again the two men to be living, and the following experiment to be performed on them.

Let the body of Reed be suddenly deprived of all its caloric, and the caloric of Edson be transferred to it; while to the body of Edson, deprived of *its* caloric, the caloric of Reed is transferred. The result of this exchange is obvious. Edson will be instantly *destroyed and fricasseed by heat*; and Reed *converted by cold into a frozen mass.*

Of these several experiments here submitted, my business is to guaranty the truth; which I deliberately do. Let candid and intelligent men conscientiously apply it, under the guidance and sanction of their knowledge and judgment, to the subject of my report, and I shall cheerfully and confidently await the issue.

The facts I have here clearly, confidently, and veritably stated, make it incontrovertibly appear, that, contrary to the notion commonly entertained respecting it, vital calorification is as little understood, and perhaps more generally and flagrantly misrepresented than any other function of living organic matter.

When therefore a chemico-physiologist presents himself to an audience as a speaker, or to the public as a writer, and gravely assures them, as Liebig, his followers and associates have long done, and as hundreds of teachers are still doing, that vital temperature is the result of *common chemical combustion*—whoever thus acts, becomes by the deed an appropriate object for

*"Scorn to point her slow unmoving finger at:"*

There are at this time, in Louisville, two females, one of them white and the other colored, weighing each three hundred and fifty pounds. They are also each about fifty-five years old; and their temperature is that of nature and health. I have carefully examined them both, and ascertained from them the following facts:

Until the age of near forty years, neither of them weighed more than one hundred and twenty-five pounds; and their diet and drink then were, in quantity and kind, the same that they are at present. And that their temperature was also the same is not to be doubted; because, as just mentioned, it is now *natural.* And it is presumable that it has been always the same; because they have been always healthy. Nor do they certainly inhale now more oxygen than they did when thirty years younger—probably not so much—their respiration being somewhat impeded and contracted by obesity. Their bulk moreover is nearly three times as large as it was in their youth. Hence, with an equal stock of calorific materials, they raise *now*, at the age of fifty-five, to the same temperature, nearly three times as much animal substance as they did at the age of twenty-five. This neither is, nor can be a deed performed by common combustion, the result of the operation of mere chemical affinity. Nor can I suppress my astonishment, that, at the middle of the nineteenth century, a period so signalized by the knowledge of nature, a contrary belief is seriously entertained by any enlightened and reflecting individual.

My astonishment on this subject would be less than it is, were facts precisely similar to those I have related, less numerous and common than I know them to be. For I doubt whether there can be found, in the United States, a single town or city containing ten or even five thousand inhabitants, where instances of a like description and import do not exist. But to conclude my report.

As a legitimate inference from the facts, illustrations, and arguments contained in this paper, I feel authorized to repeat what I have heretofore said in substance; that "VITAL ORGANIC CHEMISTRY" is a form of expression denoting nothing that actually exists. I am therefore, compelled to regard it in my best judgment, as mere *verba inana, et preterea nihil*—an empty association of words, which error and a forbidden assumption of knowledge have engendered; and which truth and genuine science disown. All which is respectfully submitted.

P. S.—In my own system a phenomenon has recently occurred, and still exists, which to my mind demonstrates no less conclusively that vital temperature is modified by an influence different from and superior to anything that belongs to the heat of common combustion.

In the great sciatic nerve I suffered recently an uncommonly violent attack of neuralgia, entirely exempt from any augmentation of vascular action, from the effects of which I have not yet recovered. In a special manner the affected limb is not a little weakened in its motive power. And, to say the least, the force and circulation of the blood in it, if not diminished, are certainly not augmented. Under these circumstances, it was natural to expect, and I did expect that the temperature of the diseased and now debilitated limb would fall below that of the sound and more vigorous one.

But not a little to my surprise, and no less to my gratification and rejoicing, the reverse is now true, and has been so since the commencement of the disease. The sound limb is coolest by at least one degree, if not by one and a half. I say that this phenomenon has occurred *to my gratification and rejoicing*." The cause of my being thus affected is sufficiently plain. The superior warmth of the diseased limb bespeaks in it a degree of vitality, which promises to minister towards restoring it to health and strength.

Whatever may be the case with regard to others, in contemplating this event, I see or fancy I see in it a striking discrepance between the production of vital temperature, and the temperature of ordinary combustion.

That I may be neither misunderstood nor misrepresented on this subject, I must, if possible be more explicit.

I do not deny that chemical forces constantly and necessarily operate and do much in the living body of man and other animals. On the contrary, I know and acknowledge that they do. But they do not operate *plastically* with respect to *living organized matter.* They neither *organize* such matter, give it figure and organic fitness, nor endow it with life. That work is performed by a higher and controlling power, denominated *vital,* which is emphatically *sui generis.*

*Addendum.*—The reader must have observed that, in my reply to Professor Liebig, I may have appeared to concur with him in the notion that the blood receives *all* its oxygen immediately from the lungs. But such is not the fact. I do not thus concur. Every article of our diet and drink contains oxygen to a greater or less amount. The blood is therefore supplied with that element through the nutritive no less certainly than through the respiratory function. And animal temperature is developed by vital action in every organ of the body, (the bones themselves not excepted) as indubitably as it is in the lungs.

Hence it was that, in my Inaugural Dissertation, dated in the year 1795 (fifty-seven years ago) I pronounced animal temperature to be "*a secretion*"—or rather a *condition of organic matter, the product of secretory action,* and its abstraction from the system by the application of cold water, cold air, or any other cold substance, to be as *genuine an evacuation* as perspiration, micturition, or the emission of blood by the puncture of a lancet. And antiquated and unfashionable as that doctrine may be now deemed, it was then, for a time, accountad orthodox in Philadelphia; and I still retain it as a sound tenet of my physiological creed. But it was not favored by Dr. Rush, because it clashed with some of his peculiar notions.

I therefore regard animal temperature as the product of a process *essentially and exclusively* VITAL. Nor do I hesitate to pronounce the chemical dogmas of the celebrated Professor of Giessen, when applied to living organic matter, whether vegetable or animal, as groundless and untenable as the wildest vagaries that ever issued from the tongue or the pen of that prince of boasters, fictionists and notion-mongers, the renowned AUREOLUS PHILLIPPUS THEOPHRASTUS BOMBASTUS DE HOENHEIM PARACELSUS.

I shall only add, that Dr. Woodhouse, a sound and strong minded thinker, and Professor of Chemistry, at the time of my graduation, in the Medical Department of the University of Pennsylvania, and one of the ablest chemists then in the United States, adopted my opinion respecting animal temperature. From the time of the publication therefore, of my Inaugural Dissertation in 1795, to the end of his life, he regularly taught the doctrine that the caloric generative of animal temperature is the product of a *bona fide secretory process*, and its passage out of the body a genuine evacuation.

To conclude, by a summary of certain sentiments I have heretofore expressed. I shall be neither surprised nor disappointed should some of the chemico-physiological sect of medical speculators cavil at and perhaps denounce my remarks on Professor Liebig's work on Animal or Organic Chemistry. I am prepared to find that such fault-finders and disparagers will be inclined to attack and condemn my strictures on that elaborate but (to me) obscure and equivocal production, because its author has not, in any part of it, clearly and fully recognized the existence of a *vital force or principle*, as an *indispensable attribute* of man, and other living organized beings, nor correctly recounted its uses and performances—because in fact he has not described it as the great *plastic power*, which, as the chief executor of the laws of heaven, for that special purpose framed and provided by heaven's supreme LAW GIVER—makes man what he is in structure, and enables him, through his endowment and aptitude, *to do what he does by his efficiency*.

Is any one of the sect referred to prepared to contend that Professor Liebig has sufficiently described and characterized the Vital principle of man and other forms of organized matter, both vegetable and animal, in all the powers and fitnesses it possesses, and in all the achievements it performs in the diversified economy it exhibits in the wide field of organized matter?

I fearlessly reply that he has not. And I with equal fearlessness defy any physiologist of any sect, or all physiologists of all sects, to specify the page or pages of his book or books, in which the Professor has thus acquitted himself—in which he has fairly and fully represented the vital principle, as possessing the numerous high, varied and important attributes it does possess, and as performing the numerous high, diversified and important functions it does perform in the economy of life and organization.

That the Professor has at certain times, and for certain purposes, spoken of the vital principle *in general terms*, with propriety and correctness, is not now, nor ever has been denied by me. But when his remarks involve *particular points and specifications*, his spirit and purport instantly and utterly change; and what he had previously attributed to *Vitality*, he then transfers to *Chemistry*.

In consequence of this versatility of purpose and conduct, his Animal or Organic Chemistry is one of the most flagrantly self-inconsistent, and self-contradictory productions I have ever examined. Hence, in the progressive improvement of the science of anthropology, and other forms of living organized matter, the popularity and influence it once possessed and exercised are as certain to pass from it, as is the life which actuates and has sustained him in a sphere so ample, attractive and commanding, is destined to pass from the mortal fabric of its distinguished author.

The two first pages of my article on "Vital Organic Chemistry" were printed off without my having examined and corrected them. In consequence of this the reader will please to make for himself the following corrections.

In t e third sentence from the top of page 104, for "humble" he will read, *simple*.

And in the fourth sentence for "apprehending and definitely comprehending," he will read,—*of penetrating* aud definitely comprehending. C. C.

## Figures and Tables

**Figure f1:**
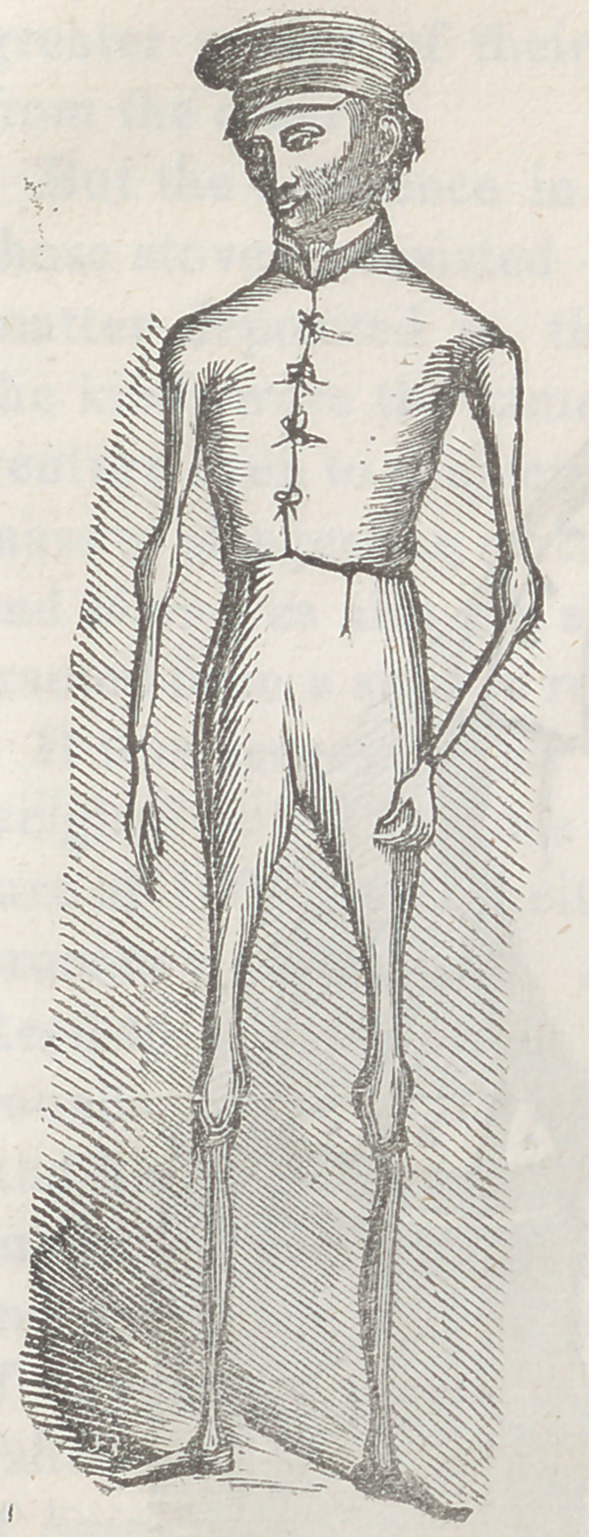


**Figure f2:**